# To the Gut Microbiome and Beyond: The Brain-First or Body-First Hypothesis in Parkinson’s Disease

**DOI:** 10.3389/fmicb.2022.791213

**Published:** 2022-03-30

**Authors:** Nathan D. Nuzum, Amy Loughman, Ewa A. Szymlek-Gay, Wei-Peng Teo, Ashlee M. Hendy, Helen Macpherson

**Affiliations:** ^1^Institute for Physical Activity and Nutrition (IPAN), School of Exercise and Nutrition Sciences, Deakin University, Geelong, VIC, Australia; ^2^Food and Mood Center, IMPACT Strategic Research Center, School of Medicine, Deakin University, Geelong, VIC, Australia; ^3^Physical Education and Sports Science Academic Group, National Institute of Education, Nanyang Technological University, Singapore, Singapore

**Keywords:** neurodegenerative, microbiota, microbiota-gut-brain axis, gut-brain-axis, gut flora

## Abstract

There is continued debate regarding Parkinson’s disease etiology and whether it originates in the brain or begins in the gut. Recently, evidence has been provided for both, with Parkinson’s disease onset presenting as either a “body-first” or “brain-first” progression. Most research indicates those with Parkinson’s disease have an altered gut microbiome compared to controls. However, some studies do not report gut microbiome differences, potentially due to the brain or body-first progression type. Based on the etiology of each proposed progression, individuals with the body-first progression may exhibit altered gut microbiomes, i.e., where short-chain fatty acid producing bacteria are reduced, while the brain-first progression may not. Future microbiome research should consider this hypothesis and investigate whether gut microbiome differences exist between each type of progression. This may further elucidate the impact of the gut microbiome in Parkinson’s disease and show how it may not be homogenous across individuals with Parkinson’s disease.

## Introduction

It has been hypothesized that Parkinson’s disease (PD) comprises two different phenotypes: a “body-first” or “brain-first” progression (Borghammer and Van Den Berge, 2019), with recent research providing some support for this hypothesis (Horsager et al., 2020; Knudsen et al., 2021). These groups are distinguished by the presence or absence, respectively, of REM sleep behavior disorder (RBD), a disorder causing individuals to actively move, sometimes violently, during REM sleep (Mckenna and Peever, 2017). This proposed dichotomy stems from research indicating that some individuals with PD develop RBD before motor symptoms (Boeve, 2010), while others do not develop RBD at all, or for whom motor symptoms precede RBD (Olson et al., 2000). The dichotomization has received criticism given the complexity and diversity of PD (Fearon et al., 2021); however, despite the dichotomy proposed, this does not preclude the possibility that these progression types could occur simultaneously (Horsager et al., 2020). Indeed, Horsager et al. (2020) state as much, outlining that the two main proposed phenotypes may not capture all phenotypic variations and that parkinsonism and RBD may arise simultaneously in cases. Regardless, this hypothesis is particularly interesting as the progression of PD is poorly understood and a topic of debate (Braak et al., 2003b; Parkkinen et al., 2008; Borghammer and Van Den Berge, 2019). Analyzing this dichotomy and applying it to future studies would help confirm or dispel the hypothesis and may allow for more targeted therapies or biomarkers to surface.

Horsager et al. (2020) found the brain-first phenotype had a different symptomatic trajectory compared to the body-first phenotype, with an overview of the differences between the two phenotypes shown in an updated paper by Borghammer et al. (2021). This difference in symptomatic trajectory was underpinned by impaired sympathetic cardiac innervation in the body-first group, like that previously reported in PD (Courbon et al., 2003; Miyamoto et al., 2006), whereas the brain-first group did not display this impairment. This suggests the body-first group was affected by misfolded α-synuclein in the enteric (ENS) and peripheral autonomic nervous systems that leads to RBD *prior* to PD diagnosis (due to the ascending progression), which aligns with Braak’s proposed staging (Braak et al., 2003a). This staging posits that progressive neurodegeneration begins peripherally *via* an external pathogen entering *via* the gastrointestinal tract and initiating α-synuclein misfolding, before advancing upward from the dorsal motor nucleus of the vagus nerve to the medulla oblongata (and other susceptible regions) before reaching the cerebral cortex (Braak et al., 2003a). It was theorized that an external pathogen entering *via* the gastrointestinal tract may begin this process, and was later revised to also include the potential entry point of the olfactory bulbs, forming the dual-hit hypothesis (Hawkes et al., 2009). However, the lack of impairment in sympathetic cardiac innervation in the brain-first group suggests a different mechanism is involved. For the brain-first group, misfolded α-synuclein could arise in the central nervous system (CNS) where motor impairments are seen first, and the descending progression of the disease could then cause RBD to develop (Horsager et al., 2020). Differences in the two phenotypes may in part explain how research both supports and refutes Braak’s proposed staging (Braak et al., 2003a,b; Jellinger, 2019). As mentioned, the body-first group perhaps aligns more with Braak’s proposed staging and the brain-first group aligns with research finding PD pathology to not follow Braak’s staging, and instead may either initiate within the CNS or may enter *via* the olfactory bulb alone and not *via* the gastrointestinal tract (Adams-Carr et al., 2016). However, given the likely involvement of the ENS in the body-first group, it is crucial to discuss the role of the microbiota-gut-brain axis, which represents the bidirectional communication network between the brain or CNS and the gut (primarily colon) or ENS (Nuzum et al., 2020). Here we discuss how the body-first or brain-first hypothesis aligns with previous PD research, particularly research focusing on the microbiota-gut-brain axis, and we propose future directions for this research.

## The Gut Microbiome and Parkinson’s Disease Progression

The impaired sympathetic cardiac activity observed by Horsager et al. (2020) in the body-first group aligns with Braak’s disease staging and gut microbiome research in PD (Braak et al., 2003b; Nuzum et al., 2020). From this perspective, α-synuclein may aggregate in the ENS before propagating up the vagus nerve and into brain regions where Lewy body formation is characteristic of PD (Holmqvist et al., 2014). This is postulated to occur through neurotropic pathogens entering *via* two routes, the nasal cavity and the gastrointestinal tract (Hawkes et al., 2009). At the gastrointestinal tract, increased intestinal permeability may allow pathogens or bacterial endotoxins to cross into the ENS to cause α-synuclein misfolding. Research has linked intestinal permeability in PD to the aggregation of misfolded α-synuclein (Forsyth et al., 2011), and tight junction proteins have been shown to be reduced in PD to controls (Clairembault et al., 2015). Additionally, local inflammation at the intestine leading to neuro-inflammation is perhaps another mechanism of PD progression (Forsyth et al., 2011; Schwiertz et al., 2018). The type(s) of bacteria present or absent in the gut may contribute to these mechanisms as outlined previously (Nuzum et al., 2020). It has been postulated that reduced abundance of short-chain fatty acid (SCFA)-producing bacteria may be crucial, as these bacteria, through the SCFA metabolites they produce, are involved in maintaining intestinal epithelial integrity and have anti-inflammatory effects (Lopetuso et al., 2013; Tan et al., 2014; Nuzum et al., 2020). Examples of such bacteria include *Faecalibacterium prausnitzii* and bacteria from the Lachnospiraceae family including *Roseburia*, which have been found to be in lower abundances in PD compared to healthy controls (Keshavarzian et al., 2015; Cilia et al., 2021; Romano et al., 2021; Shen et al., 2021).

Potential mechanisms for reduced SCFA-producing bacteria could relate to lifestyle factors including diet, physical activity, and sleep, as previously discussed (Nuzum et al., 2020). Additional mechanisms may include the use of antibiotics (Parada Venegas et al., 2019) or exposure to environmental toxins including pesticides or heavy metals which have been shown to alter the microbiome of animal models (Yuan et al., 2019). Mice exposed to chlorpyrifos had increased markers of inflammation and intestinal permeability, along with reductions in some SCFA metabolites (Zhao et al., 2016). Epidemiological evidence also shows that chlorpyrifos increases the risk of developing PD in humans (Freire and Koifman, 2012), with additional toxins indicated to be involved within PD development including various heavy metals and pesticides like rotenone (Ball et al., 2019). Supporting the potential role of environmental toxins to perturb the gut microbiome, Hill-Burns et al. (2017) found that there was increased metabolic activity of pathways involved in the degradation of herbicides, namely, atrazine, in individuals with PD compared to controls. They simultaneously showed the PD group had lower putative SCFA-producing bacteria, including bacteria from the *Lachnospiraceae* family, compared to controls, as well finding butyrate kinase, a valuable catalyst involved in butyrate synthesis, being reduced in PD.

Apart from SCFA-producing bacteria being altered, mucin-degrading bacteria including *Akkermansia muciniphila* could also be involved in PD pathology with genetic variations in mice showing the expression of this bacteria to be increased (Guardia-Escote et al., 2020), and the same genetic variation, apolipoprotein E4, also related to an increased incidence of PD in humans (Singh et al., 2014). While not commonly found to be in greater abundance across PD and gut microbiome studies, *Akkermansia* bacteria have nevertheless been reported to be in greater abundance in PD groups (Bedarf et al., 2017; Heintz-Buschart et al., 2018; Baldini et al., 2020).

Altogether, dysbiosis in PD *via* reduced SCFA-producing bacteria or through degradation of the intestinal wall is implicated in PD pathophysiology. Particularly in the body-first phenotype whereby the retrograde transmission of α-synuclein up the vagus nerve to the CNS is plausible (Holmqvist et al., 2014). Even without the vagus nerve, the involvement of the gut microbiota may still be present, as bacterial endotoxins like lipopolysaccharide and the bacteria that procures them are involved in the formation of fibrillar α-synuclein and are linked to not only GI inflammation but systemic inflammation too (Fitzgerald et al., 2019; Bhattacharyya and Bhunia, 2021).

## Inconsistencies Within Parkinson’s Disease and Gut Microbiome Literature

Gut microbiome research in PD has, however, been somewhat inconsistent with respect to microbial diversity and the differential abundance of individual bacterial taxa between patients and healthy controls (Nuzum et al., 2020). This may be due to study heterogeneity (Nuzum et al., 2020), or perhaps part of the inconsistency is due to the hypothesized brain-first and body-first phenotypes. Indeed, most studies show reduced levels of SCFA-producing bacteria, i.e., *Faecalibacterium* or *Roseburia*, in PD patients compared to controls, with some studies showing increased abundance of mucin-degrading *Akkermansia* too (Nuzum et al., 2020; Shen et al., 2021). In addition, gut microbiome differences are found even when accounting for factors known to affect the gut microbiome such as diet or gastrointestinal symptoms, like constipation (Hill-Burns et al., 2017; Wallen et al., 2020). While there are few studies accounting for lifestyle factors such as diet, this still indicates that gut microbiome differences may be driven by PD itself and may not just be an artifact of lifestyle factors or environmental differences. However, these differences may not be seen across both PD phenotypes.

## The Gut Microbiome and the Body- or Brain-First Progression and Beyond

If the body-first phenotype etiology consists of α-synuclein aggregation in the periphery before moving toward the CNS, it may be that this group has an altered gut microbiome, i.e., reduced abundance of SCFA-producing bacteria, compared to controls or to the brain-first phenotype, leading to α-synuclein aggregation through the above-mentioned mechanisms and a more inflammatory state. In contrast, in the brain-first phenotype, if α-synuclein initially aggregates within the CNS, and the periphery is not involved at the onset, then the gut microbiome differences, including reduced SCFA-producing bacteria, may not be expected, as they might not (yet) be contributing to the disease etiology in this phenotype. As an example, colonic biopsies of PD patients revealed a heterogenous expression of pro-inflammatory and glial markers indicating that only a subset of the included PD patients displayed greater inflammation within the colon (Devos et al., 2013). While there have not yet been specific studies assessing the two phenotypes and their gut microbiome composition, some research has assessed the gut microbiome of RBD in comparison to PD (Heintz-Buschart et al., 2018). Researchers found that > 75% of the operational taxonomic units that were differently abundant between the PD and the control group were qualitatively similar to the differences between RBD and control groups. Such differences included *Anaerotruncus* species, which were greater in PD and RBD compared to controls (Heintz-Buschart et al., 2018), with *Anaerotruncus* species being involved in the degradation of mucins (Raimondi et al., 2021). Additionally, *Akkermanisa* species, also putative mucin degraders (Png et al., 2010), were in greater abundances in individuals with PD who had RBD compared to individuals with PD but not RBD (Heintz-Buschart et al., 2018). While this research is limited, it is an early indicator that the body-first phenotype that aligns with Braak’s hypothesized staging may involve the gut microbiome. However, this is not to say that gut microbiome differences would not be present between controls and the brain-first group, rather that there may be particular differences between the gut microbiomes of the body-first and brain-first groups, specifically in terms of reduced SCFA-producing bacteria, which has been shown to be relatively consistent across heterogenous studies (Nuzum et al., 2020; Shen et al., 2021) or perhaps in terms of the body-first group displaying a greater abundance of putative mucin degraders like *Akkermansia*.

The two phenotypes may also help explain the variability in related research. The use of α-synuclein aggregates in the ENS as a potential biomarker for PD onset (Ruffmann and Parkkinen, 2016) may only be relevant to the body-first phenotype, considering its purported origin of α-synuclein. However, α-synuclein aggregation in the ENS may not be expected to be present in the brain-first phenotype at disease onset. Similar inconsistencies have been found when investigating constipation, which affects most, but not all individuals with PD (Fasano et al., 2015). Previous research indicates that the particular PD population (i.e., early vs. late onset) can contribute to variability in these results (Stirpe et al., 2016). The brain-first phenotype, or population group, may not present with constipation. While there was no difference between phenotype groups in terms of self-reported constipation scores (using the ROME-III), Horsager et al. (2020) did show the body-first group had greater colon transit time and greater colon volume compared to the brain-first group, indicating the body-first group displays greater instances of constipation. Previous research has stated that subjective measures of constipation are likely not as useful as direct measures of colon transit time in PD (Knudsen et al., 2017), and potential causes of delayed colon transit time may result from impaired motor activity within the ENS and the accumulation of a-synuclein within this network (Stirpe et al., 2016). If the mechanism of constipation is primarily due to impairment of the ENS, it would follow that the body-first group displays greater instances of constipation, as it is more likely that the ENS is involved in the early stages of PD progression in that phenotype than compared to the brain-first group where constipation may only occur later in disease progression. Therefore, investigations of constipation as a risk factor for PD (Adams-Carr et al., 2016), or research into α-synuclein in the ENS as a biomarker for PD (Ruffmann and Parkkinen, 2016), could acknowledge the brain- and body-first phenotypes in their research to more accurately uncover risk factors and biomarkers.

**FIGURE 1:**
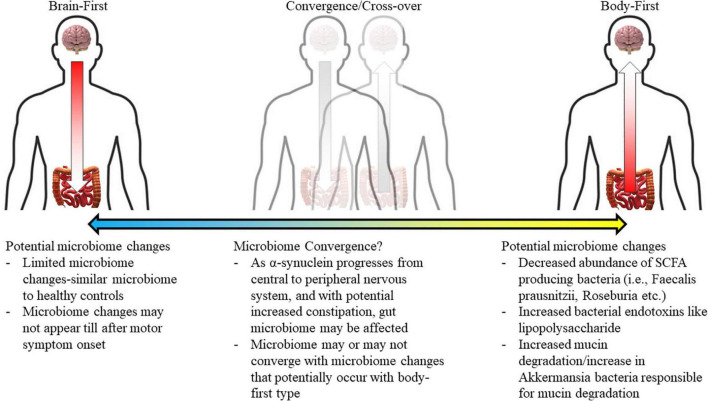
Authors’ purported hypothesis of different gut microbiomes existing for each of the brain- and body-first phenotypes. Microbiome changes are hypothesized to converge between the two phenotypes as disease progresses in each. Additionally, this is likely to occur across a gradient.

In addition to uncovering potential risk factors and biomarkers for PD, further research should look to replicate and explore the existence of the brain-first and body-first phenotypes whilst investigating their gut microbiomes. This research should simultaneously measure controls, as Horsager et al. (2020) did not statistically compare control participants with PD participants in either the brain-first or body-first group. By comparing gut microbiome composition between a control group and these phenotypes, this may help to elucidate etiological pathways of PD, depending on how the microbial composition differs between the two phenotypes. For example, based on the existing evidence, we would hypothesize that the body-first microbiome may, at first, demonstrate a decreased abundance of SCFA-producing bacteria and a reduction in bacteria known to be beneficial to intestinal permeability (Nuzum et al., 2020), whereas these specific differences may be absent in the brain-first phenotype (Figure 1). Longitudinal studies, where an initial measurement is taken at the beginning of the PD diagnosis and subsequent measures taken as PD progresses, would be particularly useful to show microbial trajectory, such as the convergence of the two groups in later disease progression as suggested by Horsager et al. (2020). The potential convergence of the two phenotypes in terms of gut microbiome composition would be supported as research shows α-synuclein transmission is possible from the CNS to the ENS (Ulusoy et al., 2017), whereby it may then cause microbiome changes similar to those seen early on in the body-first group. While the association between RBD and other neurodegenerative conditions including Alzheimer’s disease has been explored (Wang et al., 2016), Horsager et al. (2020) have not extended the discussion of the brain-/body-first hypothesis to other neurodegenerative disorders or synucleinopathies.

## Conclusion

Existing research indicates the majority of PD cases have been reported to follow the body-first approach that aligns with Braak’s hypothesized staging (Braak et al., 2003b; Kalaitzakis et al., 2008; Parkkinen et al., 2008; Zaccai et al., 2008; Beach et al., 2009). Correspondingly, Borghammer and Van Den Berge (2019) describe the polar nature of this research, which usually either provides evidence for the body-first phenotype that aligns to Braak’s staging, or evidence favoring the brain-first approach. Considering the recent findings (Horsager et al., 2020), perhaps both pathways are feasible, in which case the prevalence of each may be important to consider. From a microbiome research perspective, the existence of the two phenotypes could be confirmed, with this research potentially showing that the phenotypes have different relationships with, and importance to, the microbiome. Additionally, it is possible individuals would lie between each phenotype and that instead of this being a dichotomy, it is a continuum where both phenotypes may present simultaneously at different ratios.

Overall, Horsager et al. (2020) have presented valuable findings for this unique hypothesis that is worth considering in future microbiome-related PD research. Presently, we have identified how this hypothesis may relate to existing gut microbiome research in PD and we have provided a direction for future microbiome research and how it may further inform this hypothesis. Investigating how these potential phenotypes relate to the gut microbiome and α-synuclein pathology in the ENS may help reveal insights into PD progression and, by extension, elucidate specific treatment opportunities.

## Data Availability Statement

The original contributions presented in the study are included in the article/supplementary material, further inquiries can be directed to the corresponding author/s.

## Author Contributions

NN was involved in the conception of the article. All authors were involved in writing the manuscript.

## Conflict of Interest

The authors declare that the research was conducted in the absence of any commercial or financial relationships that could be construed as a potential conflict of interest.

## Publisher’s Note

All claims expressed in this article are solely those of the authors and do not necessarily represent those of their affiliated organizations, or those of the publisher, the editors and the reviewers. Any product that may be evaluated in this article, or claim that may be made by its manufacturer, is not guaranteed or endorsed by the publisher.

## Abbreviations

ENS: enteric nervous system
PD: Parkinson’s disease
RBD: REM sleep behavior disorder
SCFA: short chain fatty acid.
